# The influence of affective touch on interoceptive and exteroceptive sensory integration in infants: evidence from heartbeat-evoked and event-related potentials

**DOI:** 10.3389/fpsyg.2025.1603183

**Published:** 2025-10-13

**Authors:** Yukari Tanaka, Masako Myowa

**Affiliations:** Graduate School of Education, Kyoto University, Kyoto, Japan

**Keywords:** affective touch, interoception, exteroception, Heartbeat-evoked potentials (HEP), event-related potentials (ERP), multissensory integration

## Abstract

**Introduction:**

The daily accumulation of multimodal interactions with a primary caregiver is thought to integrate interoceptive and exteroceptive information in the infant brain, which may contribute to the development of social cognition. However, the neural mechanisms underlying this integration in infancy remain largely unexplored. Focusing on affective touch as a sensory stimulus affecting infants' interoception during caregiver-infant interactions, this study examined how the perception of affective touch (i.e., stroking) affects the neural processing of interoceptive-exteroceptive integration in infants' brains.

**Methods:**

During the exposure phase, infants' legs were stroked while viewing a stranger's face (Affective Touch, AT), while another face was presented without touch as a control (No-Affective Touch, No-AT). In the test phase, infants viewed the same faces in isolation. Electroencephalography (EEG) and electrocardiogram (ECG) were simultaneously measured to compare two neural indices between conditions in the test phase: heartbeat-evoked potentials (HEPs), reflecting interoceptive-exteroceptive integration, and event-related potentials (ERPs) to faces, indicating exteroceptive processing.

**Results:**

Results showed that affective touch enhanced HEPs in frontal-central regions and modulated ERPs to faces, with stronger P400 amplitudes in parietal regions. In addition, a positive correlation between HEP responses and ERPs to faces emerged, with individual differences in resting HEP, reflecting baseline interoceptive-exteroceptive sensitivity, being associated with HEP modulation during the test phase.

**Discussion:**

These findings suggest the role of multisensory experiences, particularly those involving touch, in enhancing interoceptive-exteroceptive integration, with individual variations. Our findings support the hypothesis that affective caregiver-infant interactions facilitate interoceptive-exteroceptive integration, a fundamental process underlying the development of social cognition, and emotional regulation.

## 1 Introduction

To ensure survival and adaptation in dynamic and unpredictable environments, mammals, including humans, rely on a dynamic process of internal states known as allostasis, which allows for anticipatory adjustments to external conditions (McEwen and Wingfield, 2003; Sterling, 2012). For example, when a threat is anticipated, heart rate increases in preparation for a possible escape or confrontation. However, during early development, infants lack the neurophysiological maturity required to independently regulate allostasis (Gao et al., 2015). Consequently, they rely on primary caregivers to regulate their internal states. Caregivers support infants by feeding them when hungry, holding them to regulate body temperature, and using touch to stabilize heart rate and sleep–wake cycles (Bauer et al., 1997; Feldman, 2007; Feldman et al., 2002). These daily interactions with caregivers involve multisensory inputs through which infants gradually develop the capacity to regulate their own internal states.

To receive effective care from a specific person, infants need to accurately identify their primary caregiver. This identification relies on the infant brain's ability to determine whether multisensory stimuli are consistently associated with the caregiver. Recent research highlights the central role of interoceptive-exteroceptive integration in infancy for the construction of a neural representation of the primary caregiver (Atzil et al., 2018). Interoception refers to internal physiological states, such as heart activity and hunger, whereas exteroception encompasses external sensory inputs, including auditory, visual and tactile stimuli (Craig, 2003; Damasio, 2003). Through repeated multisensory experiences—such as soothing touch and familiar faces during feeding—infants gradually associate specific exteroceptive cues with the regulation of their internal states. This interoceptive-exteroceptive integration facilitates the development of a neural construct of the caregiver as a source of comfort and security, laying the foundation for social cognition (Atzil et al., 2018).

As a multisensory stimulus, affective touch plays a unique and essential role in the integration of interoceptive and exteroceptive signals, particularly in caregiver-infant interactions. Affective touch uniquely bridges exteroceptive and interoceptive processing by providing tactile sensations of external contact while simultaneously influencing internal physiological states such as heart rate coordination (Tanaka and Kanakogi, 2021; Zoltowski et al., 2022). Affective touch, including slow and gentle stroking, is most observed in caregiving contexts, particularly during soothing interactions (Feldman, 2011; Ferber et al., 2008). For example, slow and gentle stroking has been shown to positively affect infants' physiological states by decreasing heart rate and increasing heart rate variability (Van Puyvelde et al., 2019). These physiological effects are thought to be mediated by C-tactile afferent fibers, a type of nerve fiber that responds selectively to slow, gentle touch (McGlone et al., 2014). C-tactile afferents transmit signals from the skin to subcortical areas and cortical regions, including the anterior insula (Morrison and Croy, 2021), which is a core region involved in the integration of interoceptive and exteroceptive signals (Gu et al., 2013; Kleckner et al., 2017). This pathway suggests that affective touch serves as a powerful means for infants to connect external sensory cues with internal regulatory effects. However, it remains largely unexplored how experiences of multisensory input involving tactile cues (i.e. stroking) influence the integrated processing of exteroceptive and interoceptive signals in the infant brain, particularly during early development.

In order to examine the mechanisms by which the infant brain integrates interoceptive and exteroceptive information, heartbeat-evoked potentials (HEPs) and event-related potentials (ERPs) serve as key neurophysiological markers. HEPs index the integration of interoceptive and exteroceptive signals, measured as neural activity time-locked to the R-peak of the electrocardiogram (Coll et al., 2021; Park and Blanke, 2019). By contrast, ERPs capture neural responses time-locked to external sensory events, such as faces. Through repeated multisensory exposure, the brain learns to associate these inputs, enabling predictive processing, in which one sensory input facilitates anticipation of another (Banellis and Cruse, 2020; Den Ouden et al., 2009; Shams et al., 2011; Tanaka et al., 2018). Visuo-tactile input may facilitate multisensory integration, allowing the infant brain to anticipate tactile sensations (e.g., being touched) and interoceptive states (e.g., heart rate stabilization in response to soothing touch) solely from perceiving another's face. For example, the mere visual presentation of emotional faces amplified HEP responses of 5-month-old infants (Maister et al., 2017). These findings suggest that early multimodal inputs accompanied by touch may play a pivotal role in promoting interoceptive–exteroceptive integration in infancy.

It is important to acknowledge the potential interplay between interoceptive-exteroceptive integration and exteroceptive multisensory integration. For instance, research conducted on adults has demonstrated a correlation between HEP amplitudes and ERP responses to exteroceptive signals (Marshall et al., 2017; Ono et al., 2024), suggesting that interoceptive-exteroceptive integration enhances the processing of exteroceptive stimuli. Aguirre et al. (2019) found that when 9-month-old infants observed their caregiver's face while being gently stroked on the foot, their heart rates stabilized—a response that was absent when the face belonged to a stranger. This finding suggests that the experience of heartbeat stabilization through stroking while gazing at the caregiver's face in daily caregiving situations may facilitate the integration of both visuo-tactile and interoceptive information of infants. To gain a more profound understanding of the relationship between visuo-tactile (exteroceptive-exteroceptive) integration and visuo-interoceptive (exteroceptive-interoceptive) integration, the interplay between HEP and ERP responses should be addressed.

Furthermore, although it has received little attention, it is imperative to investigate the neural mechanisms underlying individual differences in how affective touch influences interoceptive and exteroceptive sensory integration. For instance, Maister et al. (2017) observed that infants exhibiting stronger heart-beat-evoked potential (HEP) responses demonstrated a behavioral preference for visual stimuli that were synchronized with their heartbeat, as opposed to non-synchronized stimuli (Maister et al., 2017). This sensitivity to heartbeat synchrony may reflect an enhanced capacity for interoceptive-exteroceptive integration. The potential relationship between these individual differences and the impact of affective touch on infants' interoceptive-exteroceptive integration process, as evidenced by HEP responses, merits further exploration. However, the investigation of these specific neurophysiological factors remains limited. Recent research has demonstrated that resting HEP amplitude in adults can reflect individual differences in interoceptive processing. Studies have found that adults with certain anxiety disorders exhibit different resting HEP amplitudes compared to healthy controls (Pang et al., 2019). While infants are in the initial phases of developing interoceptive-exteroceptive integration, resting HEP may serve as a putative marker of baseline sensitivity to such integration. Rather than representing a stable trait, it should be regarded as an exploratory indicator that may provide preliminary insight into individual variability during early development.

Based on the above, the primary objective of this study was to investigate the impact of visuo-tactile sensory experiences (i.e., observing others' faces while undergoing tactile stimulation) on subsequent neural integrative processes in early infancy. Specifically, the study compared two conditions, based on a previous behavioral study (Longa et al., 2019): in the exposure phase, an affective touch (AT) condition, where infants experienced gentle stroking while looking at a stranger's face, and a no-touch (No-AT) condition. Subsequently, in the test phase, we measured infants' electroencephalograms (EEGs) and electrocardiograms (ECGs) simultaneously while the infants looked at the same face without tactile input. We measured both heartbeat-evoked potentials (HEP) and event-related potentials (ERP) as neurophysiological markers of interoceptive-exteroceptive and exteroceptive-exteroceptive sensory integration, respectively. The present study will address the relationship between HEP and ERP, thereby elucidating the interrelatedness of interoceptive-exteroceptive and exteroceptive-exteroceptive integration in infants' brains. As a secondary objective, we investigated the relationship between resting-state HEP, which serves as an index of individual differences in the sensitivity to the integration of interoception-exteroception, and the effect of affective touch on HEP and ERP.

We hypothesized the following three: (i) HEP responses, reflecting interoceptive-exteroceptive integration, would be enhanced in the AT condition compared to the No-AT condition, suggesting that affective touch may facilitate the infant brain's exteroceptive-interoceptive integration. (ii) ERP responses to faces would be more pronounced in the AT condition, indicating that tactile-visual simultaneous input strengthens infants' exteroceptive tactile-visual integration processing. (iii) The amplitude of HEP responses to faces would be correlated with ERP amplitude, indicating that the facilitation of interoceptive-exteroceptive integration is related to visuo-tactile integration.

We also examined the relationship between individual differences in the resting HEP amplitude, measured before the task, and the HEP and ERP responses in the test phase to explore whether baseline capacity for exteroceptive-interoceptive integration (resting HEP) is related to the effect of touch on exteroceptive-interoceptive integration in the test phase of the experiment.

## 2 Materials and methods

### 2.1 Participants

Forty-five full-term Japanese infants (22 boys; *M* = 216 days, *SD* = 46.3; range = 153–276 days) aged 5–9 months, without clinical risk or diagnosis, participated in the experiment. Participants were recruited from a research registry maintained by our laboratory. Of these, data from 12 infants (4 boys and 8 girls) were excluded for the following reasons: fussiness during the exposure phase (*n* = 3); failure to complete the entire test phase due to crying (*n* = 2); technical errors during the exposure phase (*n* = 3); excessive noise in the EEG data (*n* = 4). Sample size was determined based on previous research that investigated infants' HEP during perception of emotional faces (Maister et al., 2017). However, we did not conduct a formal power or sensitivity analysis, and we acknowledge this as a methodological limitation. Informed consent was obtained from the parents of the participants before the study was conducted. The study was approved by the Kokoro Science Ethics Committee in Kyoto University (No. 28–p−3).

### 2.2 Stimuli

#### 2.2.1 Face stimuli in the exposure and test phase

**Figure 1:**
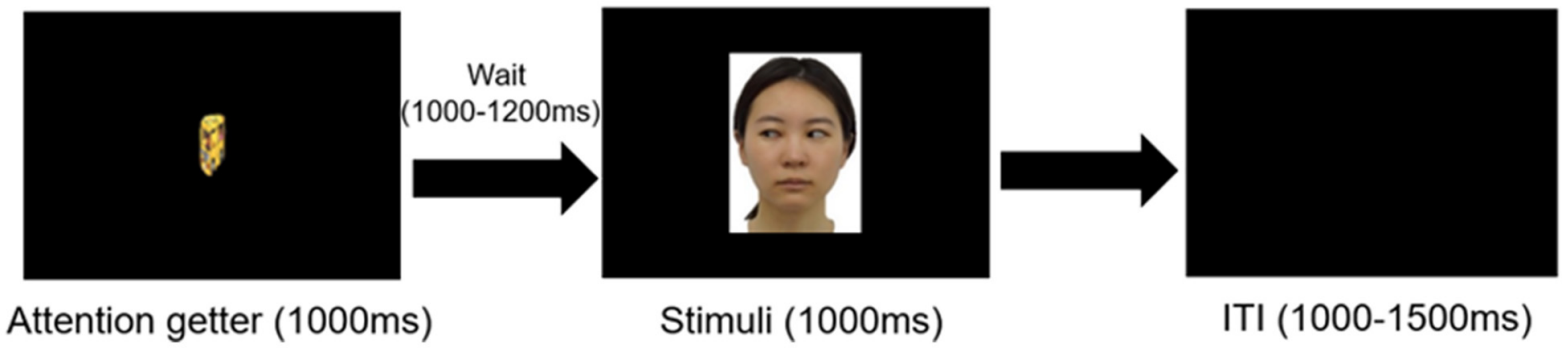
Experimental procedure in the test phase. Only visual stimuli were presented without touch. Face stimuli corresponding to the exposure phase, presented for 1,000 ms following about 3 sec of attention-grabbing animation (attention getter) and a blank screen. Inter-trial intervals varied between 1,000–1,500 ms.

Visual stimuli were faces of two unfamiliar female adults. Both wore plain clothing with hair tied back and bangs secured to the sides. They faced the front with a neutral expression, and their gaze was directed to the right or left (Figure 1). The averted-gaze stimuli were prepared to reduce the effect of direct gaze to facilitate face learning, based on previous behavioral research (Longa et al., 2019). Stimulus size and brightness were controlled by software (adobe Photoshop, Adobe Systems Inc. California, U.S.). The size of the each image was approximately 21° × 14° when viewed from a distance of 60 cm according to previous ERPs study to faces in infancy (Elsabbagh et al., 2012; Farroni et al., 2004). Since two gaze directions (right and left) were taken for the two women, a total of four different stimuli were prepared.

#### 2.2.2 Video stimuli in the resting phase

For the resting-state measurement, we used a 120–s video with sound, containing no human or animal figures, following the prior research (Anderson and Perone, 2018). The video comprised four 30–s scenes, featuring visuals such as a swinging soap bubble and a floating geometric figure. To validate the neutrality of the stimuli, six adults (mean age = 22.80, three males) blind to the purpose of this study rated their arousal and emotional valence, respectively, using a 5-point scale (1: calm to 5: awake for arousal; 1: negative to 5: positive for valence). The mean arousal score was 2.80 (SD = 0.52) and the emotional valence score was 3.30 (SD = 0.12).

### 2.3 Procedure

The experiment consisted of the following three phases; a resting state phase, an exposure phase (during which infants perceived face stimuli with or without stroking), followed by a test phase (during which infants only saw the face stimuli). Before the resting phase, an EEG cap was attached on the infants' heads, and ECG electrodes were attached on the infant's chest and abdomen. The infant's ECG and EEG were measured during the resting phase, exposure phase and the test phase (See also **EEG and ECG Data Acquisition and Processing** for details). The resting state measurements were taken first to avoid the possible influence of affective touch on the infant's internal state. The all phases took place in a shielded room. Infants are seated on a blanket (60 cm × 40 cm × 2 cm) that lay on the lap of the caregiver to minimize the effect of the mother's cardiac activity on the infant's heartbeat.

#### 2.3.1 Resting phase

Infants sat on the mother's lap and watched an approximately 2–min video without music on a 43-inch monitor (EX-LD4K431DB, I-O DATA, Ishikawa, Japan) at a distance of about 65–70 cm from the infant. The mother was instructed not to look down and not to interact with their infants, so that the infants' attention would remain directed toward the neutral video stimuli without additional social cues from the caregiver. When the infant became inattentive to the video, an attention getter with sound was presented in the center of the screen. As soon as it was confirmed that the infant's attention was again directed to the display, the video was restarted. Soon after the resting phase, exposure phase was conducted.

#### 2.3.2 Exposure phase

Prior to the exposure phase, a table (60 cm × 47 cm × 67 cm) was positioned in front of the infant to obstruct their view of their own feet. An experimenter sat under the table, out of the infant's sight. Mothers were instructed to keep their gaze lowered and not to interact with their infants, in order to minimize the influence of maternal gaze on infants' attention to the face stimuli and to prevent the tactile manipulation performed under the table from being visible to the infant. To monitor the infant's gaze direction and body movements, video cameras were placed—one mounted atop the screen (C615 HD webcam; Logitech, Newark, CA) and another positioned diagonally to the left behind the infant (HDR XR502V, SONY, Tokyo, Japan).

First, an attention-getter was presented on the screen for a few seconds until infants looked at it. Once infants looked at the attention-getter, the face of one of the two women with an averted gaze was presented for 5 sec on the monitor. When one face was presented, the experimenter, who was hidden under the table, stroked the infant's foot twice with a soft brush at a rate of 2–3 cm/s (Affective Touch condition, AT condition). The stroking velocity corresponded to the optimal range for activating C-tactile (CT) afferents (Löken et al., 2009; McGlone et al., 2014). Previous studies have shown that even short episodes of stroking (i.e., 3–5 s) at this velocity are sufficient to elicit CT activation and measurable physiological effects in infants (Van Puyvelde et al., 2019). To avoid the carry-over effect of touch on the next trial, a 5–s inter-trial interval was implemented. Subsequently, the attention-getter was shown again and the face of the other female model was presented. At this time, the experimenter did not touch the infant's feet (No-Affective Touch condition, No-AT condition). Each face stimulus was presented alternately. The experimenter switched the left and right feet to be touched for each trial in the AT condition. The gaze direction of visual stimuli was consistent across conditions (i.e., when a face in the right gaze direction was presented in the AT condition, another face in the same gaze direction was also presented in the No-AT condition). One face was consistently paired with the Affective Touch (AT) condition and the other with the No-Affective Touch (No-AT) condition. EEG and ECG analyses were conducted based on these condition-specific pairings. The combination of the face (2 models) and condition (AT or No-AT), the order in which the face pictures were presented (AT first or No-AT first), and the first order in which the left and right sides of the infant's feet were touched (Left or Right) were counterbalanced across participants.

The exposure phase finished after infants either (i) completed 7 trials per condition (14 trials in total), or (ii) became inattentive to the experimenter, as indicated by their showing fussiness or large body movements. The mean number of presentation trials was 5.36 (range = 5–7, SD = 0.92), lasting about 4–5 min. When infants became fussy, started crying, or did not pay attention to the screen after less than 10 trials in total, the experiment was terminated at that stage and no further data analysis was performed (*n* = 3). To confirm that infants paid attention to the screen, we analyzed the video off-line and calculated the amount of time the infant was looking at the screen. The mean-looking time for infants was 3.55 s (SD = 1.34, the proportion of the looking time was 71.0%) in the AT condition and 3.42 s (SD = 1.29, the proportion of the looking time was 68.4%) in the No-AT condition. There was no significant difference in the duration between conditions with paired *t*-test (*t* = 1.22, *p* = 0.65). For each retained trial, video recordings were inspected offline to confirm that infants were looking at the screen during the presentation of the visual-tactile stimulus, and not at their mother. Trials in which infants looked away from the screen or directed their gaze toward the mother were excluded from further EEG/ECG analyses.

#### 2.3.3 Test phase

After the exposure phase, the experimenter left the shielded room, and the partitioned table used in exposure phase was removed in order to ensure that the infants' attention was sustained on the on-screen face stimuli. Caregivers were instructed not to interact with infants during the recording. The EEG and ECG recording started again once the infants sat still. The experimental procedure is shown in Figure 1. Following simple attention-grabbing animation (1,000 ms) and subsequent blank screen (1,000–1,200 ms), Two types of face pictures, identical to those presented in the exposure phase, were presented for 1,000 ms. The inter-trial interval was 1,000–1,500 ms. The stimuli in the AT and No-AT conditions were presented alternately to prevent repetition suppression (Grill-Spector et al., 2006). When infants' attention deviated during recording, we paused the experiment and presented short 30–s videos unrelated to the experiment were shown Recording resumed once the infants redirected their gaze to the screen. The session continued until the infants either lost interest or completed a total of 100 trials, lasting approximately 5 min in total. All stimuli were presented using E-Prime software (Psychology Software Tools, Inc., Pittsburgh, PA).

### 2.4 EEG and ECG data acquisition

We measured EEG with a 64-channel Geodesic Sensor Net and analyzed using Net Station software (Electrical Geodesics, Inc., Eugene, OR) sampled at 1,000 Hz. Impedance was measured before EEG recording and kept below 50 kΩ. Electrodes were placed according to the 10–10 international system for electrode placement. All recordings were initially referenced to the vertex and later re-referenced to the average of all channels. We recorded ECG using a physiological amplifier Polyam-ECGIIA (Nihon-Santeku Co. Ltd., Osaka, Japan) with Ag/AgCl electrodes that were placed on infants' chest and stomach in a 3-lead ECG setup, to monitor their cardiac activity throughout the task. The out-put signals were connected to the Polygraphic Input box of the EEG amplifier (EGI Inc.). We also recorded infants' behavior during data acquisition using 2 video cameras (HDR XR502V, SONY, Tokyo, Japan; C615 HD webcam; Logitech, Newark, CA) to check motion artifacts.

We measured EEG and ECG during the resting phase, exposure phase and test phase. Our main focus was the effect of affective touch on infants subsequent neural processing, HEP and ERPs to faces in test phase were included as main analysis. The EEG during exposure phase and ECG data was not reported here because of the motion artifact and facial myoelectricity associated with facial expressions including smiles and frowns. We coded emotional expression at behavioral level during the stimulus presentation (see **2.5.2 Behavioral coding during exposure phase**).

### 2.5 Data analysis

#### 2.5.1 EEG data processing

Off-line EEG pre-processing was performed using BrainVision Analyzer software (Brain Products, Munich, Germany). In the resting phase, resting-HEP were analyzed, and in the test phase, HEPs and ERPs to faces were analyzed.

**HEP**: EEG data were digitally filtered offline using a 0.3–30 Hz band-pass filter. An Independent Component Analysis (ICA) was performed on raw EEG data to remove eye-movement artifacts and Cardiac Field Artifacts for HEP (CFA) (Terhaar et al., 2012). Using ICA to remove CFAs is highly effective in removing cardiac artifacts from the EEG signal (Luft and Bhattacharya, 2015; Park et al., 2014; Terhaar et al., 2012). Bad channels were replaced using topographic interpolation. The data was then re-segmented to extract the HEPs, with a duration of 600 ms (−200 to 400 ms) time-locked to the R-Peak (Maister et al., 2017). Artifacts were screened with the following automatic detection methods: eye blinks (140-mV threshold in the frontal region within 80 ms post-stimulus presentation), eye movement (55-mV threshold), and excessive noise (i.e., channels with amplitudes exceeding 200 mV were excluded). We also inspected all EEG data visually and marked bad channels. Segments with 10 or more bad channels were excluded. Additionally, after recording, a third experimenter checked the infants' body movements off-line. It was checked whether infants perceived stimuli while keeping still (coded as “0”), they moved their body a little (coded as “1”), or markedly (coded as “2”) per each trial. Upon visual screening analysis of this video recording data, segments containing marked body movements (i.e., coded as “2”) were also excluded from averaging, as were those segments that were likely to be due to motion artifacts.

Mean valid trial numbers were 244.88 R-peaks in the resting phase (range = 83–336, SD = 52.08), and in the test phase, 32.23 trials for the AT condition (range = 11–67, SD = 14.37) and 32.13 trials for the No-AT condition (range = 11–63, SD = 14.07). We confirmed that the number of retained trials did not significantly differ between the AT and No-AT conditions (*t*(31) = 0.13, *p* = 0.897). Participants with fewer than 10 valid trials were excluded (*n* = 2 in the test phase). The averages of amplitudes were calculated separately for resting phase, and condition in the test phase (the AT condition and the No-AT condition), 200–50 ms recording prior to stimuli presentation used as the baseline period to avoid artifacts from the R wave rising edge (Canales-Johnson et al., 2015). In the present study, 150–300 ms time windows (P200) were determined based on the previous study (Maister et al., 2017).

To determine the target regions in test phase, six frontal-to-central regions were included based on the previous study for infants (Maister et al., 2017). In addition, to examine the relationship between HEP and ERP to faces, additional 3 parietal regions (left, middle, and right parietal regions) were included as regions or interests based on the previous literature (Halit et al., 2003; Leppänen et al., 2007). Total 9 different regions were determined (right frontal, middle frontal, left frontal, right central, middle central, left central, right temporo-parietal middle parietal and left temporo-parietal), which included 3–5 channels per each region, by visual inspection of the ERP wave form at each electrode, to improve the signal-to-noise ratio (for the electrode sites analyzed in this study, see Supplementary Figure 1).

**ERPs to faces in the test phase**: To examine the relationship between HEP and exteroceptive information processing (hypothesis 2), we analyzed ERPs with face stimuli as the onset. EEG data were digitally filtered off-line using a 0.3–30 Hz band-pass filter. The data was segmented into a 1,000 ms epoch that was time-locked to the onset of the face stimulus (target), preceded with a 200 ms pre-stimulus baseline period (so a total of 1,200 ms) based upon prior infant research investigating EEG components in the perception of the face (De Haan et al., 2003; Kooijman et al., 2009; Leppänen et al., 2007). The procedure of artifact rejection was the same as that of HEP preprocessing. We used, on average, 18.84 trials for the AT condition (range: 10–38), 19.32 trials for the No-AT condition (range: 10–38) per infant. Participants with fewer than 10 good trials were excluded (*n* = 4).

The averages of amplitudes were calculated separately for each condition (the AT condition and the No-AT condition) the 200 ms recording prior to stimuli presentation used as the baseline period. The time windows of target ERP components were determined based on a previous study (Leppänen et al., 2007) that measured ERPs to faces in infants of overlapping age in months with the present study: N290 (200–320 ms) and P400 (280–480 ms). The target regions were the same as those of HEPs described above (for the electrode sites analyzed in this study, see Supplementary Figure 2).

#### 2.5.2 ECG analysis in the resting measurement

Recent studies have reported significant associations between heart rate variability (HRV) and heartbeat-evoked potentials (HEPs), suggesting that interindividual differences in autonomic regulation may be linked to cortical processing of cardiac signals (Luft and Bhattacharya, 2015; Park and Blanke, 2019). In addition to the analysis of the resting HEPs, exploratory correlation analyses were conducted to examine the relationship between HRV indices in the resting phase (e.g., SDNN, RMSSD, LF/HF) and (1) resting-state HEP amplitudes, and (2) HEPs and P400s amplitudes during the test phase. These analyses aimed to evaluate whether variability in autonomic function is associated with individual differences in interoceptive and exteroceptive brain responses. The detailed procedure for calculating heart rate and heart rate variability from the ECG data is provided in the Supplementary materials.

#### 2.5.3 Behavioral coding during exposure phase

We also conducted exploratory coding of infants' emotional expressions during the exposure phase to examine individual differences in the effect of stroking on HEP and ERP responses to faces in the test phase. It is possible that afferent interoceptive signals may facilitate integrated processing in the brain in infants who had greater emotional responses to stroking. The experimenter coded 1(Very negative) to 5 (Very positive) for each trial, based on the following criteria. Very negative; infants cried or twisted his/her body to show refusal, Negative; infants showed fussiness, and raised an eyebrow, Neutral; infants showed no specific emotional expression, Positive; infants smiled or neutral utterance watching toward the face stimuli, Very positive; infants showed laugh, or reached out against facial stimuli. 25 % of all data was also coded by the other person who did not know the purpose of the study. The coding reliability (Copper coefficient) between the two experimenters was 0.88 in Touch condition and 0.83 in No-Touch condition. The mean emotional score was calculated in each condition. Given the modest number of participants and trials, this analysis should be regarded as exploratory, and its statistical power is limited. Therefore, the results are interpreted cautiously and considered supplementary to the main neurophysiological findings.

### 2.6 Statistical tests

According to Hypothesis 1 and 2, we tested whether the significant effect of condition (AT condition or No-AT condition) on HEPs and ERPs to faces was a two-tailed cluster-based permutation test (Maris and Oostenveld, 2007), using a Monte Carlo simulation. Using this method, all samples that showed a significant (*p* < 05) relationship with our independent variable were clustered according to spatiotemporal adjacencies, and cluster-level statistics were calculated by taking a sum of the *t*-values for each cluster. A Monte-Carlo permutation method then generated a *p*-value by calculating the probability that this cluster-level statistic could be achieved by chance, by randomly shuffling and resampling the independent variable structure a large number of times (2,000 repetitions) (Maris and Oostenveld, 2007). Spatiotemporal clusters that had a resulting Monte-Carlo corrected *p*-value of less than the critical alpha level of 0.05 were interpreted as significant. We chose N290 and P400 for ERPs to faces, as well as P200 for HEPs as time domain, and 9 regions as spatial domain.

## 3 Results

### 3.1 HEP in the test phase

**Figure 2:**
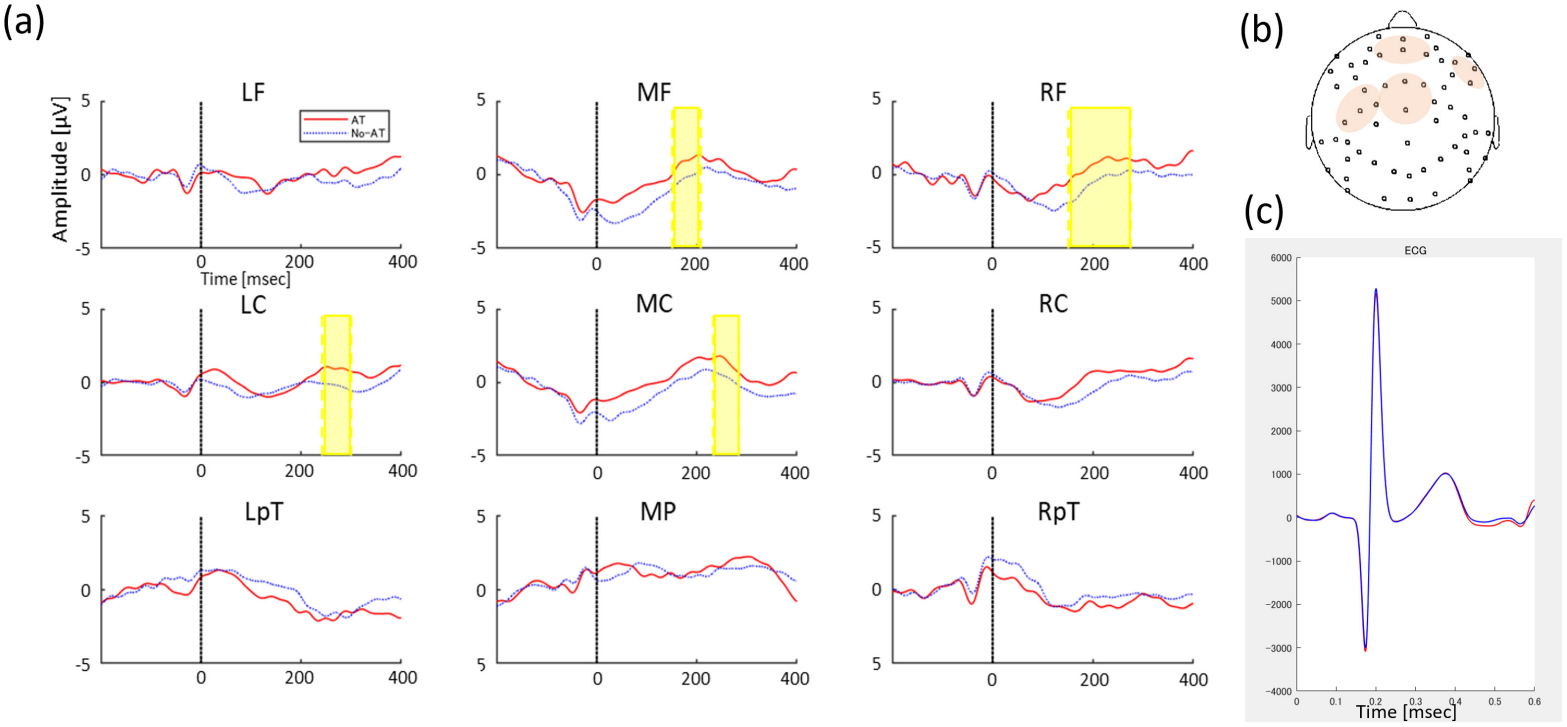
**(A)** Grand-average HEP between AT condition (Red solid line) and No-AT condition (Blue dot line) in 9 regions (left frontal, LF; middle frontal, MF; right frontal, RF; left central, LC; middle central, MC; right central, RC; left temporo-parietal, LTp; middle parietal, MP; and right temporo-parietal, RTp). The y-axis indicates amplitude magnitude [μV] and the x-axis indicates time (ms), where 0 refers to the time of the heartbeat R peak and the time series of HEP changes from then until 400 ms. Significant differences in HEP between conditions are indicated by highlighted periods. **(B)** ROIs including channels where significant HEP between conditions, and **(C)** Average ECG signal between conditions (red line represents AT condition and blue line represents No-AT condition) in the test phase.

We found significant differences between conditions in the frontal-central clusters approximately 150–300 ms from the ECG R peak, with more positive potential activity in the AT condition than in the No-AT condition (*p* = 0.005, *T*_sums_ = 651.44, middle frontal: 15–208 ms, right frontal: 151–275 ms, left central: 242–300 ms, middle central: 233–284 ms, Figure 2A, 2B). An identical analysis was conducted on the ECG channel, which did not identify any significant temporal clusters discriminating between conditions, confirming that the effect was specific to the cortical processing of the heartbeat and not due to cardiac field artifacts (Figure 2). To examine potential baseline influences of the cardiac R-peak on the EEG, we conducted additional cluster-based permutation test during the baseline window. This analysis revealed no significant condition differences, confirming that the observed post-R effects cannot be attributed to baseline activity.

### 3.2 ERPs to faces in the test phase

**Figure 3:**
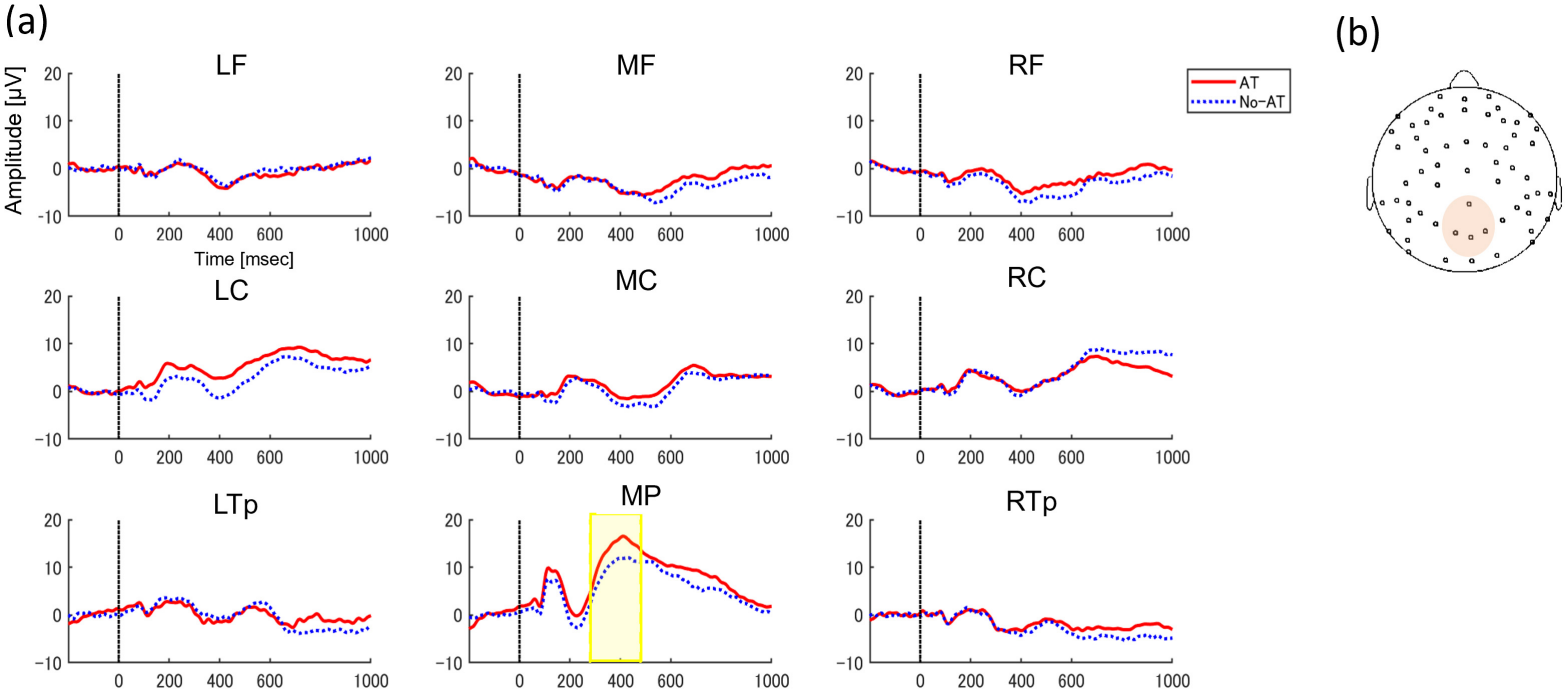
**(A)** Grand-average ERPs to faces between AT condition (Red solid line) and No-AT condition (Blue dot line) in 9 regions (left frontal, LF; middle frontal, MF; right frontal, RF; left central, LC; middle central, MC; right central, RC; left temporo-parietal, LTp; middle parietal, MP; and right temporo-parietal, RTp). The y-axis indicates amplitude magnitude [μV] and the x-axis indicates time (ms). 0 refers to the time of face stimulus presentation. Significant differences in ERPs between conditions are indicated by highlighted periods. **(B)** ROIs including channels where significant ERPs between conditions.

In N270 time window, three clusters were automatically classified, but no clusters showed significant differences in amplitudes between conditions (AT or No-AT, *p*s > 0.09). In P400 time window, five different clusters were classified, and we found greater positive amplitudes in AT condition compared to No-AT condition in parietal clusters (*p* = 0.036, *T*_sums_ = 691.05, middle parietal: 280–480 ms, Figure 3).

### 3.3 The relationship between HEP and ERPs during the test phase

**Figure 4:**
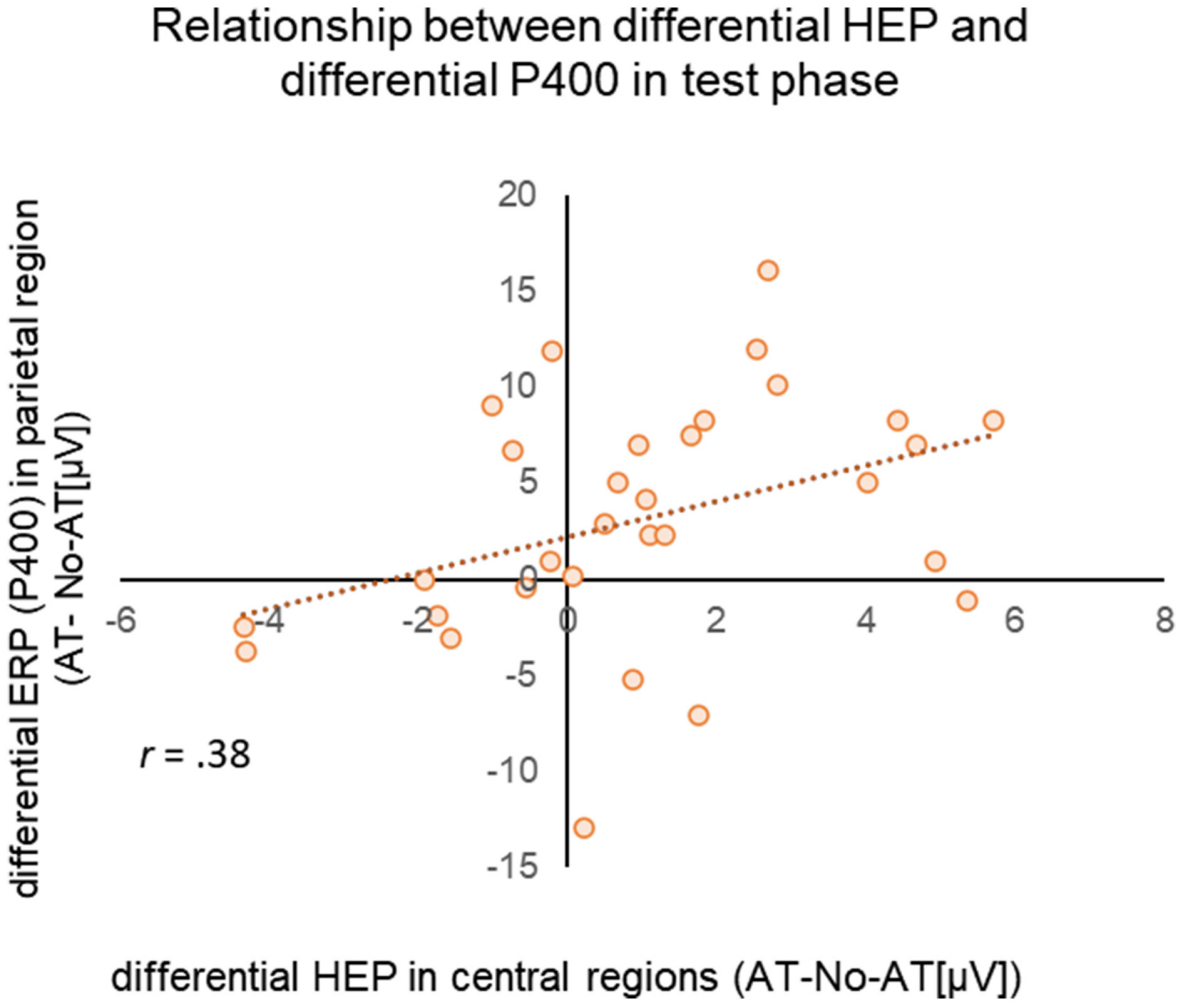
Scatterplot of differential HEP (AT/No-AT) (x-axis) for the test phase HEP in the middle central region and differential ERP (AT/No-AT) (y-axis) in the middle parietal region during the test phase. The dotted line represents the regression line.

We predicted that HEP amplitudes in response to faces would correlate with ERP amplitudes, supporting the hypothesis that affective touch facilitates interoceptive–exteroceptive and visuo-tactile integration (Hypothesis 3). To test this, we calculated the differential amplitude (AT condition—No-AT condition) for HEP (P200) and ERP (P400) after stimulus onset. Pearson correlation analysis revealed a significant positive correlation (*r* = 0.38, *p* = 0.035) between the difference in HEP amplitude in the central region (150–300 ms latency) and the difference in P400 amplitude in the parietal region (Figure 4). On the other hand, no significant correlation was found between the difference in HEP amplitude in the frontal region and the difference in P400 amplitude in the parietal region (*r* = 0.128, *p* = 0.493).

### 3.4 Relationship between resting-HEP and HEP in test phase

We examined the relationship between HEP responses during the test phase and resting HEP to explore potential individual differences in how infants' brains integrate interoceptive and exteroceptive sensory information. This analysis focused on HEP in the resting state, building on previous findings in adults that suggests HEP amplitude at rest reflect the integration of interoceptive and exteroceptive signals and vary across individuals (Pang et al., 2019; Schmitz et al., 2021; Schulz et al., 2013).

**Figure 5:**
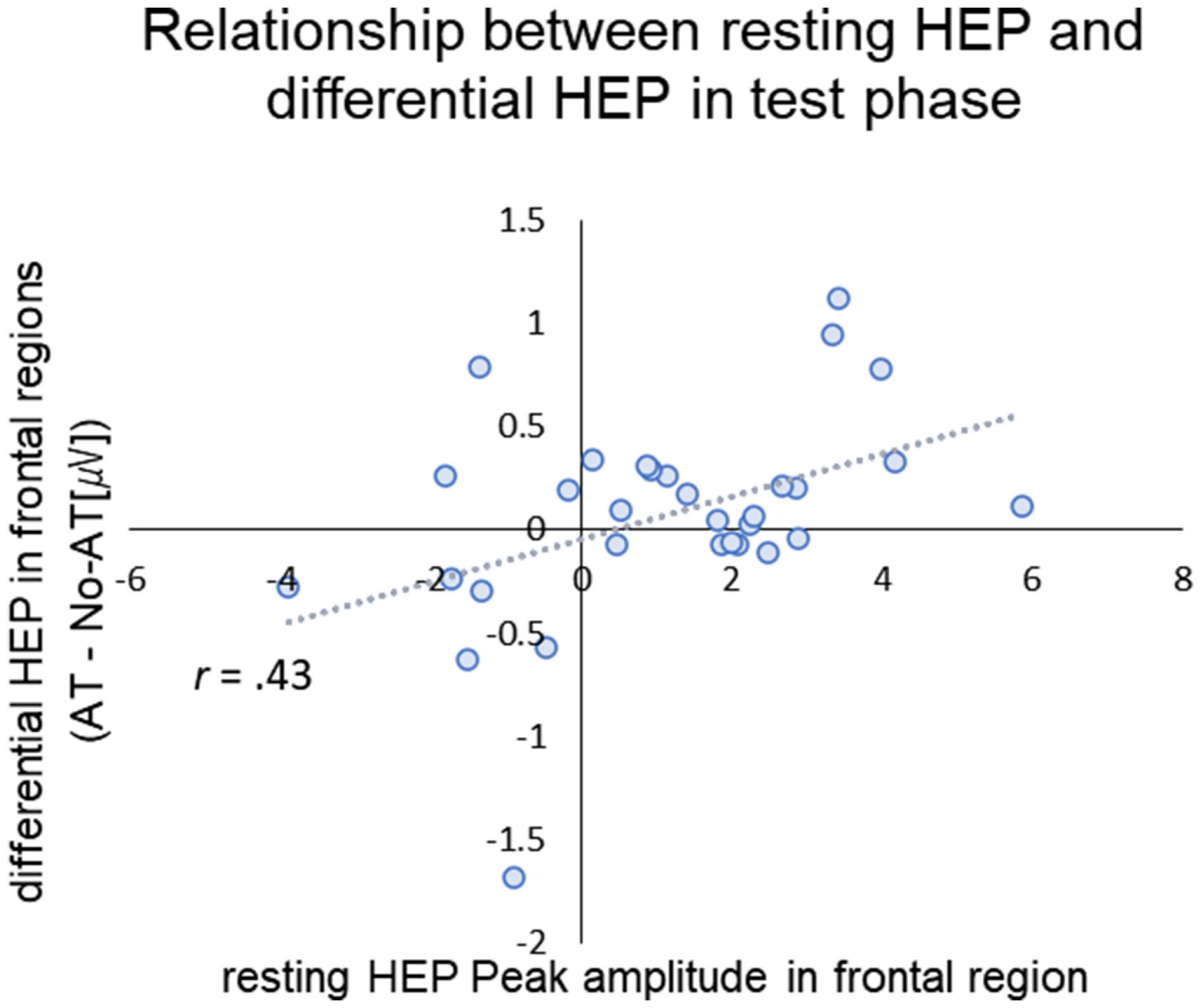
Scatter plot of the peak amplitude of resting-HEP in frontal region (x-axis) and differential HEP (AT- No-AT) in frontal region in the test phase (y-axis). The dotted line and gray area represent the regression line.

For resting HEP, positive peak amplitudes were calculated for frontal (4 medial frontal channels labeled restHEP-Frontal) and central (9 contralateral channels, labeled restHEP-Central) positive peak amplitudes (see Supplementary Figure 3 for channel settings). We selected the areas based on a previous study, which suggested that HEP deviations occurred in the fronto-central regions (Coll et al., 2021). For test phase, we calculated the mean differential amplitude between conditions (AT—No-AT [μV]) over frontal (mean amplitude between MF and RF) and central regions (mean amplitude between LC and MC), corresponding to the time when significant difference between conditions. Pearson's correlation analysis showed that the magnitude of the resting HEP in frontal regions was positively correlated with the difference in HEP in frontal regions during the test phase (*r* = 0.434, *p* = 0.045, with Holm corrections) (Figure 5). Given the modest effect size, this association should be interpreted as preliminary. The magnitude of resting HEP in the central region was not significantly associated with HEPs during the test phase (Frontal: *r* = 0.275, *p* = 0.135, Central: *r* = 0.268, *p* = 0.145, uncorrected). There was also no significant relationship between resting HEP and P400 differential potential during the test phase (restHEP-Frontal: *r* = −0.046, *p* = 0.81; restHEP-Central: *r* = 0.215, *p* = 0.245, uncorrected).

We also conducted correlation analyses to examine the relationships between resting-state HRV indices—including mean RRI, SDNN, RMSSD, and LF/HF ratio—and (1) HEP amplitudes in the frontal-central region during rest, and (2) HEP and ERP (P400) differential amplitudes during the test phase (AT—No-AT [μV]). However, none of the resting-state heart rate or HRV indices showed significant correlations with any of the HEP or ERP measures (−0.37 < *r*s < 0.21, *p*s > 0.05 uncorrected).

### 3.5 Relationship between emotional display in exposure phase and HEP in test phase

We exploratory examined the correlation analysis of the emotional display in exposure phase and HEP in test phase. For test phase, we calculated the mean differential amplitude between conditions (AT—No-AT [μV]) over frontal (mean amplitude between MF and RF) and central regions (mean amplitude between LC and MC), corresponding to the time when significant difference between conditions. Pearson's correlation analysis showed that no significant correlation between emotional display score and HEP differences between condition (0.01 < *r*s < 0.28, *p*s > 0.05, uncorrected).

## 4 Discussion

This study examined how multisensory experiences—such as observing another person's face while perceiving affective touch—affect infants' neural processing of interoceptive and exteroceptive integration. Infants aged 5–9 months were exposed to two conditions: observing a stranger's face while their legs were stroked (affective touch condition) and observing a different face without tactile stimulation (no-affective touch condition). Neurophysiological responses were assessed through heartbeat-evoked potentials (HEPs) and event-related potentials (ERPs), elicited while perceiving the same visual stimuli without tactile input. To elucidate individual differences in sensory integration, the relationship between resting-state HEPs and task-related HEPs was analyzed.

We identified four key findings. First, after exposure to affective touch (AT), viewing a stranger's face significantly enhanced HEP responses in fronto-central regions compared to the No-AT condition, supporting Hypothesis 1. Sec, AT during exposure influenced subsequent ERP responses to faces, with stronger parietal P400 amplitudes in the AT condition than in the No-AT condition, supporting Hypothesis 2. Third, the magnitude of the AT effect on central HEP correlated with its effect on ERPs to faces (P400 differential amplitude), suggesting that simultaneous tactile (affective touch) and visual (faces) input enhance exteroceptive processing, supporting Hypothesis 3. Forth, individual differences in resting HEP amplitudes in frontal regions were associated with how affective touch influenced interoceptive-exteroceptive integration, as reflected in differential HEP amplitude during the test phase.

The finding that seeing a stranger's face while being stroked influenced infants' subsequent HEP during face perception may reflect the interoceptive-exteroceptive integration in the infant brain. Recent adult neuroscience studies have identified HEP as an index of interoceptive-exteroceptive integration (Banellis and Cruse, 2020; Engelen et al., 2023; Sel et al., 2017). For instance, when auditory feedback was synchronized with an adult participant's heartbeat, omitting part of the auditory feedback elicited greater HEP, suggesting that the omission violated the anticipation of the auditory stimulus. A similar effect is likely to have occurred in this study. During the exposure phase, infants were presented with a stranger's face in conjunction with tactile stimulation, potentially enhancing interoceptive-exteroceptive integration. In the subsequent test phase, when only the face was presented, the absence of interoception-associated tactile stimuli led to an expectation violation, resulting in a heightened HEP response.

Viewing a stranger's face during stroking also modulated infants' parietal P400 responses, possibly reflecting multisensory integration. Studies in adults show P400 is involved in multisensory associative learning (Cai et al., 2018; Liu et al., 2011). Cai et al. (2018) reported that P400 was enhanced by accuracy training in sound localization, where participants were exposed to audiovisual stimuli. A study with infants found that after exposure to a novel word as an auditory stimulus accompanied by tactile input, later presentation of the same word without touch enhanced EEG responses related to multisensory integration (Tanaka et al., 2018). Our findings can similarly be interpreted as associative learning of visual-tactile multisensory information during AT exposure, reflected in increased P400 in the test phase.

Notably, the magnitude of the effect of affective touch on HEP in the central region was associated with that of P400 in the parietal region. This finding aligns with adult studies showing a relationship between HEP and ERP amplitudes in response to exteroceptive sensory stimuli, such as visual and auditory inputs (Marshall et al., 2017; Ono et al., 2024). The positive correlation between HEP and P400 amplitudes suggests that infants more sensitive to interoceptive-exteroceptive integration also excel at integrating multisensory exteroceptive information. Biologically, this relationship can be explained by predictive coding mechanisms (Friston, 2010; Friston and Kiebel, 2009). The brain continuously generates predictions about sensory inputs based on internal states, where accurate predictions through interoceptive-exteroceptive integration enhance exteroceptive sensory processing (Seth, 2013; Seth and Friston, 2016) to minimize surprise and optimize behavioral responses. In this study, due to the absence of affective touch in the test phase, infants with larger prediction errors of the interoceptive signal inferred from exteroceptive input (i.e., visual input only) may have detected greater prediction errors for the absence of tactile stimuli, reflected in the correlation between HEP and P400 amplitude. The present findings may be interpreted within the predictive coding framework, suggesting that infants may integrate interoceptive and exteroceptive information to reduce prediction errors. However, this interpretation should be considered a hypothesis rather than a confirmed mechanism. To clarify this hypothesis, more research is needed.

In addition, infants' resting HEP was correlated with their frontal HEP response during the test phase. The underlying mechanisms mediating this relationship remain to be elucidated; however, studies of clinical adult populations have suggested a correlation between resting HEP amplitude and emotion (Pang et al., 2019; Schmitz et al., 2021). For instance, patients diagnosed with generalized anxiety disorder (GAD) have been observed to exhibit larger resting open-eye HEP amplitudes compared to typical adults (Pang et al., 2019). This finding has been interpreted as reflecting hypersensitivity in GAD patients to interoceptive sensory information when processing exteroceptive sensory stimuli. In infancy, resting HEP should be considered a exploratory marker, rather than a stable trait measure. While it may provide preliminary insight into individual variability in interoceptive-exteroceptive integration, its functional role remains unclear even in adult research, underscoring the need for cautious interpretation and further longitudinal investigation.

While we also examined the relationship between resting-state HRV indices and neural measures (HEP and ERP), no significant associations were observed. One possible explanation is that HRV variability was limited in our relatively healthy infant sample, thereby reducing statistical sensitivity. Another possibility is that HRV may capture different aspects of autonomic regulation that are not directly reflected in cortical measures of interoceptive–exteroceptive integration. Future studies using larger and more heterogeneous samples may clarify whether HRV contributes to individual variability in sensory integration.

The present findings enhance our understanding of the relationship between perceptual and emotional development in early infancy through tactile sensory experiences. While affective touch is known to influence infants' heart rate, respiration (Aguirre et al., 2019; Van Puyvelde et al., 2019), and behavioral responses (Ferber et al., 2008; Jean and Stack, 2009; Tanaka and Kanakogi, 2021), this study provides novel evidence of its role in interoceptive-exteroceptive integration, as reflected in infants' HEPs. Recent work has further emphasized the neurobiological underpinnings of affective touch, highlighting the role of C-tactile afferents and their projection to the insular cortex in shaping emotional regulation and social bonding (La Rosa et al., 2024). These findings reinforce the idea that affective touch provides a powerful neurobiological pathway through which multisensory caregiver–infant interactions can scaffold early socio-emotional development. Given the high sensitivity of the early postnatal brain to environmental factors (Knudsen, 2004; Nelson and Gabard-Durnam, 2020), understanding how affective touch shapes neural development is crucial for elucidating the link between social interaction and brain maturation in infancy.

Our findings also suggest that infant HEPs may not merely reflect an afferent neural response to interoceptive signals but also serve as a predictive mechanism for integrating interoception and exteroception based on multisensory input. A comparison of research on HEP in infants with that in adults reveals a paucity and inconsistency of studies in the field (Maister et al., 2017; Weijs et al., 2023). For instance, Weijs et al. (2023) found no HEP differences in 5–7-month-old infants exposed to visual stimuli synchronized or unsynchronized with their heartbeat, possibly due to insufficiently salient stimuli. In contrast, the present study demonstrates that experiencing visuo-tactile input (face and stroking) during exposure facilitates interoceptive-exteroceptive integration in infants. These findings underscore the crucial role of affective touch in shaping neural mechanisms underlying the development of caregiver representation through daily multisensory interactions (Atzil et al., 2018).

This study has some limitations. The observed effects may not be specific to affective touch, as both affective and stimulating touch (e.g., poking or tickling) influence infants' arousal states and could consequently impact HEP. However, including both conditions within the same subject is impractical, as they significantly alter arousal levels. An alternative approach would be to vary tactile stimulation speed, though longer exposure times may be required for infants to associate faces with tactile stimuli. Our preliminary experiments showed that infants' attention to face stimuli decreased after prolonged exposure, increasing the risk of disengagement during the test phase. Future research should explore affective touch effects in dynamic social contexts, as HEP is sensitive to motion artifacts, necessitating advancements in measurement and analysis techniques to study HEP in naturalistic parent-infant interactions.

Another methodological consideration concerns the regional distribution of our findings and possible baseline influences. Although visual inspection suggested differences in the MF and MC regions at baseline, statistical analyses confirmed that no significant condition differences were present during this period. Thus, the observed MF and MC effects in the post-R window cannot be attributed to baseline activity but should be interpreted as heartbeat-related modulations. However, its magnitude appears sensitive to baseline treatment and should therefore be interpreted cautiously as a heartbeat-aligned modulation rather than a purely evoked effect. Importantly, condition differences emerged in medial frontal (MF) and medial central (MC) regions, whereas lateral central regions (e.g., RC) did not show significant effects. One possible explanation is that interoceptive–exteroceptive integration is preferentially mediated by midline cortical structures, including medial prefrontal and cingulate regions, which are consistently implicated in interoceptive processing in adults. Furthermore, the alternation of stroking between left and right legs was implemented to balance hemispheric effects. While this procedure helped to avoid systematic lateralization, it may also have contributed to stronger medial rather than lateral activation. Future work directly comparing unilateral and bilateral stimulation could clarify whether contralateral or hemispheric-specific contributions are masked by this procedure.

Finally, an important limitation is that we used unfamiliar faces as visual stimuli. The rationale for this choice was that using the mother's face would have introduced variability that could not be controlled, given that infants differ greatly in the amount and type of tactile experiences they receive from their mothers in daily caregiving contexts. By employing novel faces, we were able to minimize the influence of prior tactile experience. Nevertheless, from an ecological validity perspective, it is crucial for future studies to compare responses to the mother's face (or other familiar caregivers' faces) with those to unfamiliar faces. Such a design would allow researchers to disentangle how everyday caregiving experiences shape interoceptive–exteroceptive integration, providing a more comprehensive understanding of the role of familiarity in affective touch processing.

In summary, this study examined how affective touch influences interoceptive-exteroceptive integration in the infant brain. EEG and ECG were recorded in infants aged 5–9 months to assess how neural responses to faces are shaped by prior affective touch. During exposure, infants viewed a stranger's face either with stroking or without touch. In the test phase, they viewed the same faces without touch, allowing comparison of neural responses based on prior exposure. HEPs and ERPs were measured to evaluate the impact of affective touch. Resting HEPs were analyzed to examine individual differences in interoceptive-exteroceptive integration. Findings revealed that prior affective touch enhanced HEP responses in central regions and ERP responses (P400) in parietal regions during face perception. A positive correlation emerged between HEP and ERP (P400) responses. Additionally, individual differences in resting HEP amplitudes correlated with frontal HEP responses. These results highlight that affective touch facilitates interoceptive-exteroceptive integration in infants, with individual variability, supporting the role of daily multisensory input in sensory integration in infants' brain.

## Data Availability

The datasets presented in this article are not readily available because to make the data from this study publicly available, an opt-out procedure must be conducted through the ethics committee. For inquiries regarding potential secondary use of the data, please contact the corresponding author. Requests to access the datasets should be directed to Yukari Tanaka, tanaka.yukari.2z@kyoto-u.ac.
