# Supplementary material for: The influence of affective touch on interoceptive and exteroceptive sensory integration in infants: evidence from heartbeat-evoked and event-related potentials
Source: Front Psychol. 2025 Oct 13;16:1603183. doi: 10.3389/fpsyg.2025.1603183 (PMC12554756; doi:10.3389/fpsyg.2025.1603183)
Supplement: Supplementary file 1 [file Data_Sheet_1.pdf]

Supplementary Material

1 Supplementary Figures and Tables

Supplementary Table 1.
Descriptive statistics of heart rate and heart rate variability during resting-state measurement. Mean, standard deviation, minimum, and maximum values are reported for each parameter. HRV indices are presented in milliseconds (ms) or squared milliseconds (ms^2) as appropriate.

	Mean	SD	Min	Max
Mean RRI	456.78	34.77	392.82	518.58
SDNN	30.57	10.06	11.89	52.41
RMSSD	22.73	11.19	4.24	50.51
LF	234.22	152.74	41.72	701.34
HF	191.26	167.02	8.88	679.13
LF/HF	2.11	1.64	0.17	5.35

Supplementary Figures

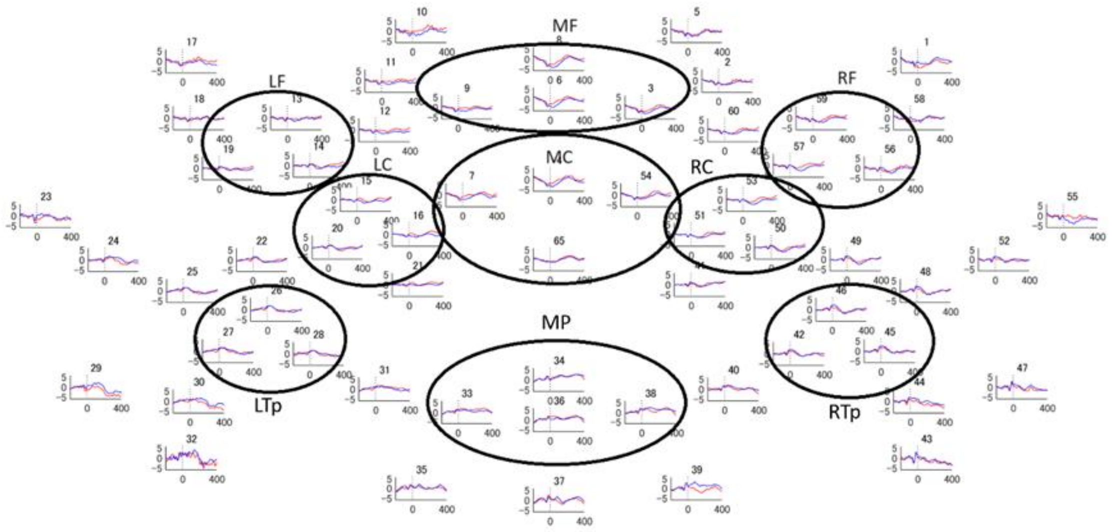

Supplementary Figure 1. Grand average HEP and target regions in test phase. LF: left-frontal, MF: middle-frontal, RF: right-frontal, LC: left-central, MC: middle-central, RC: right-central, LTp: left-temporoparietal, MP: middle parietal, and RTp: right-temporoparietal.

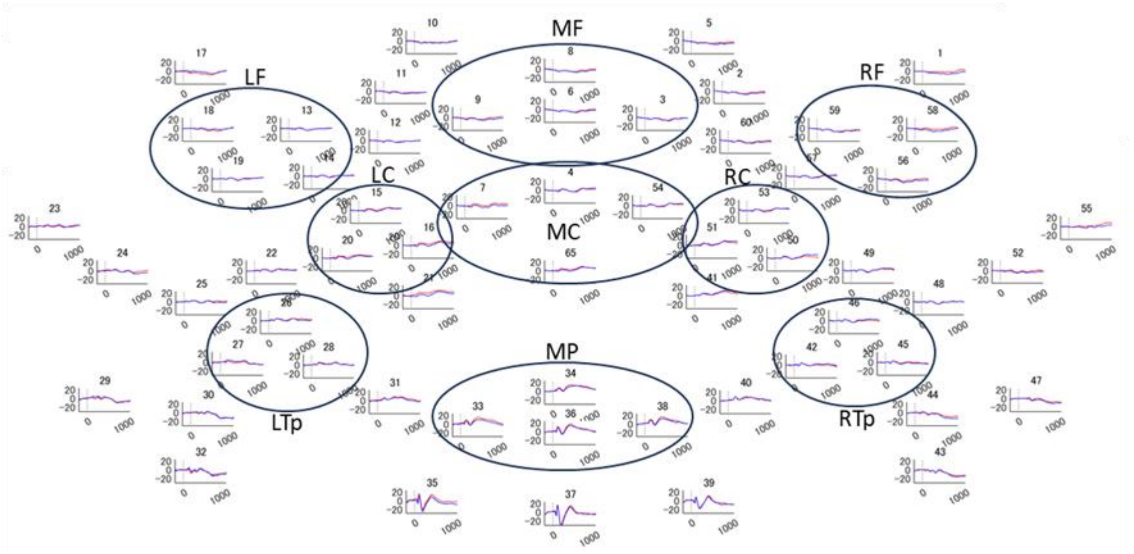

Supplementary Figure 2. Grand average ERP to faces and target regions in test phase. LF: left-frontal, MF: middle-frontal, RF: right-frontal, LC: left-central, MC: middle-central, RC: right-central, LTp: left-temporoparietal, MP: middle parietal, and RTp: right-temporoparietal.

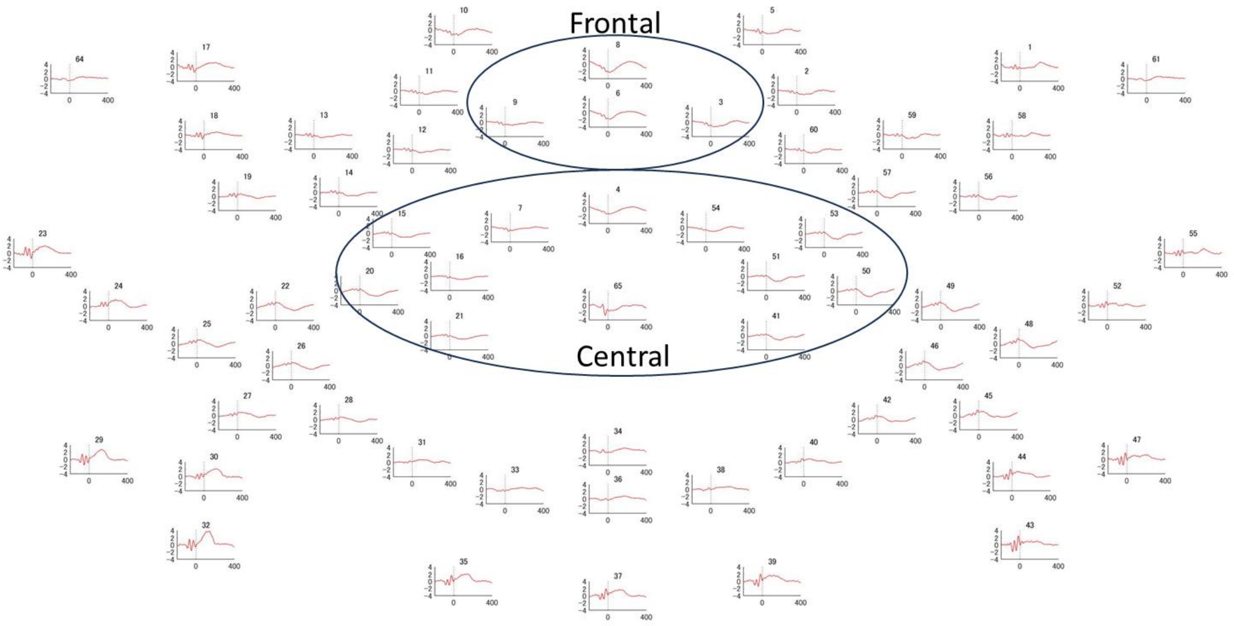

Supplementary Figure 3. Grand average ERP to faces and target regions at rest. Based on the HEP peak, Frontal and Central regions were determined.
